# The BRCA1/2-directed miRNA signature predicts a good prognosis in ovarian cancer patients with wild-type BRCA1/2

**DOI:** 10.18632/oncotarget.2963

**Published:** 2014-12-11

**Authors:** Yunyan Gu, Mengmeng Zhang, Fuduan Peng, Lei Fang, Yuanyuan Zhang, Haihai Liang, Wenbin Zhou, Lu Ao, Zheng Guo

**Affiliations:** ^1^ College of Bioinformatics Science and Technology, Harbin Medical University, Harbin, China; ^2^ Key Laboratory of Ministry of Education for Gastrointestinal Cancer, Department of Bioinformatics, Fujian Medical University, Fuzhou, China; ^3^ Department of Pharmacology, Harbin Medical University, Harbin, China; ^4^ Department of Obstetrics and Gynecology, the Second Affiliated Hospital of Harbin Medical University, Harbin, China

**Keywords:** miRNAs, BRCA1, BRCA2, Prognosis, Ovarian Cancer

## Abstract

Ovarian cancer patients carrying alterations (i.e., germline mutations, somatic mutations, hypermethylations and/or deletions) of *BRCA1* or *BRCA2* (*BRCA1/2*) have a better prognosis than *BRCA1/2* alteration non-carriers. However, patients with wild-type *BRCA1/2* may also have a favorable prognosis as a result of other mechanisms that remain poorly elucidated, such as the deregulation of miRNAs. We therefore sought to identify BRCA1/2-directed miRNA signatures that have prognostic value in ovarian cancer patients with wild-type *BRCA1/2* and study how the deregulation of miRNAs impacts the prognosis of patients treated with platinum-based chemotherapy. By analyzing multidimensional datasets of ovarian cancer patients from the TCGA data portal, we identified three miRNAs (hsa-miR-146a, hsa-miR-148a and hsa-miR-545) that target *BRCA1/2* and were associated with overall survival and progression-free survival in patients with wild-type *BRCA1/2*. By analyzing the expression profiles and Gene Ontology functional enrichment, we found that carriers of *BRCA1/2* alterations and patients with miRNA deregulation shared a common mechanism, regulation of the DNA repair-related pathways, that affects the prognosis of ovarian cancer patients. Our work highlights that a proportion of patients with wild-type *BRCA1/2* ovarian cancers benefit from platinum-based chemotherapy and that the patients who benefit could be predicted from BRCA1/2-directed miRNA signature.

## INTRODUCTION

Ovarian cancer is one of the most fatal malignancies among women, with high-grade serous ovarian adenocarcinoma being the leading cause of death[1, 2]. The annual incidence of ovarian cancer varies by geographical area and by age worldwide[3]. The majority of ovarian cancer patients succumb to the disease within 5 years of diagnosis[4]. Approximately 80% of ovarian cancer patients are responsive to treatment with platinum-based drugs. However, most patients will relapse and become resistant to subsequent therapy[5, 6].

*BRCA1* and *BRCA2* are the key genes involved in the DNA damage response and DNA repair processes, mainly via homologous recombination[7]. *BRCA1* and *BRCA2* work in concert to protect the genome from double-strand DNA damage during DNA replication. *BRCA1* and *BRCA2* mutations in ovarian cancers, are associated with defects in homologous recombination and genomic instability. Current researches have demonstrated a trend towards favorable outcomes in patients who are deficient in *BRCA1* and *BRCA2* compared with patients with wild-type *BRCA1* and *BRCA2* as a result of the DNA damage induced by platinum-based chemotherapy[8, 9]. *BRCA*-defects include germline mutations, somatic mutations, hypermethylations and copy number deletions. Unfortunately, *BRCA*-deficiency most likely occurs in only 30% of ovarian cancer patients[8, 10-12].

MiRNAs are small non-coding RNAs (~ 22 nucleotides) that regulate target gene expression by blocking the translation or degradation of their target mRNAs. Deregulation of miRNAs is involved in the initiation and progression of ovarian cancer[13]. MiRNAs participate in a variety of biological processes, such as the immune response, as well as proliferation and metastasis, which are hallmarks of cancer[14-16]. The aberrant expression of miRNAs in cancers indicates their potential to act as oncogenes or tumor suppressor genes[17]. Additionally, miRNA expression patterns have been associated with the prognosis of ovarian cancer[18, 19]. For example, the increased expression of miR-25 has been closely associated with the poor prognosis of epithelial ovarian cancer[20], indicating that miR-25 may serve as a predictive biomarker for the prognosis of epithelial ovarian cancer. Moreover, miRNAs have been shown to participate in the DNA repair pathway by regulating *BRCA1*/2 during carcinogenesis. Moskwa P *et al.* reported that the over-expression of miR-182 inhibited the expression of the *BRCA1* protein and affected homologous recombination-mediated repair[21]. Sun C *et al.* reported that miR-9 mediated the down-regulation of *BRCA1* and impeded DNA damage repair in ovarian cancer[22].

In this paper, the genes *BRCA1* and *BRCA2* are abbreviated as *BRCA1/2* for clarity. *BRCA1/2*-deficient patients subjected to platinum-based treatment have significantly improved survival compared with the patients with wild-type *BRCA1/2*[9]. However, only a small proportion of ovarian cancers exhibit direct genetic or epigenetic alterations in *BRCA1/2*. By contrast, the deregulation of miRNAs is an alternative mechanism that may affect the expression of *BRCA1/2* and further regulate the DNA damage response and DNA repair pathways. Herein, we hypothesized that ovarian cancer patients with wild-type *BRCA1*/2 but with miRNA deregulation may also have better prognosis than patients with wild-type *BRCA1*/2 but no deregulation of miRNA. Our work sought to identify miRNAs that could predict the prognosis of ovarian cancer patients who had no alterations in *BRCA1*/2 and had initially been treated with a platinum-based chemotherapy regimen. Our hypothesis was that these miRNAs could impede DNA damage repair by reducing *BRCA1*/2 expression, thereby increasing the sensitivity of cancer cells to chemotherapy. We identified that the up-regulation of three miRNAs (hsa-miR-146a, hsa-miR-148a and hsa-miR-545), which target *BRCA1*/2, in patients with wild-type *BRCA1*/2 was associated with good overall survival (OS) and progression-free survival (PFS), a finding that has important implications for the clinical management of ovarian cancers.

## RESULTS

### Survival differences between patients with and without alterations in *BRCA1/2*

Here, 317 high-grade serous ovarian adenocarcinomas, together with information on mRNA expression, miRNA expression, mutation, promoter methylation, DNA copy number and patient clinical characteristics, were identified in the TCGA data portal[2] (Table 1). Bolton KL *et al.* reported that *BRCA1* alteration carriers and *BRCA2* alteration carriers show similar survival patterns[9]. Thus, we combined the data from patients with somatic or germline mutations, copy number alterations and/or hypermethylations in *BRCA1* or *BRCA2* into the group “*BRCA1/2* alteration carriers”. *BRCA1* and *BRCA2* were altered in 69 and 33 samples, respectively (Supplementary Table 1). Three samples had both *BRCA1* and *BRCA2* alterations. The 99 *BRCA1/2* alteration carriers had a significantly better OS (*P*=3.00E-03; 49.5 vs 41.9 months; log-rank test; Supplementary Figure 1) than the 218 patients with wild-type *BRCA1/2*.

**Table 1:** Clinical features of ovarian cancer patients

Characteristic	TCGA data
**Age** (median 59.58, range 27.21-87.47)	
<=59.58	155
>59.58	154
**Stage**	
II	15
III	249
IV	52
**Grade**	
II	24
III	286
**Response Status**	
CR*	75
Non-CR*	187
**Subtype**	
Proliferative	89
Mesenchymal	74
Differentiated	92
Immunoreactive	62

* CR depicts Complete Response. Non-CR depicts non-complete response, including partial response, stable disease and progressive disease.

### Identification of prognostic miRNAs in *BRCA1/2* wild-type ovarian cancers

The 218 wild-type *BRCA1/2* ovarian cancers were randomly assigned to a training set (n=109) and a testing set (n=109). The training set was used to detect prognostic miRNAs. Fifty-seven miRNAs that are predicted to target the genes *BRCA1* or *BRCA2* were included in the analysis. According to a univariate Cox proportional hazards regression analysis, 3 of the 57 miRNAs were significantly associated with OS in patients with wild-type *BRCA1/2*. We calculated a three-miRNA (hsa-miR-146a, hsa-miR-148a and hsa-miR-545) signature risk score for each patient (see *Methods*). Using the median risk score as the cutoff, patients were classified into a miRNA-related high-risk group and a miRNA-related low-risk group. The patients with low-risk scores were expected to have better survival outcomes. As a result, the miRNA-related low-risk group had longer median OS and PFS than did the miRNA-related high-risk group (*P*=4.80E-04, median OS= 52.2 vs 26.9 months; Figure 2A).

**Figure 1:**
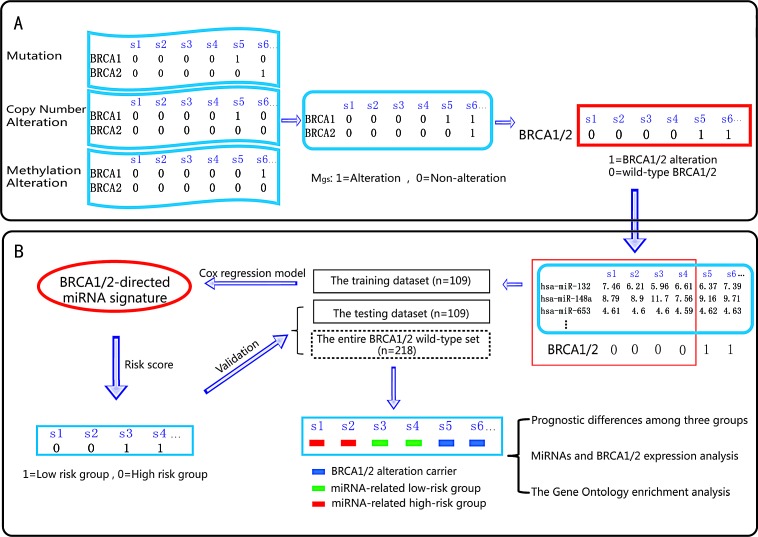
Schematic overview of our analysis procedure A. By integrating the mutation profile, copy number variation profile and methylation alteration profile, the alteration profile (*M_gs_*) was built: the columns reflect ovarian cancer samples, and the rows reflect genes. If a gene (*g*) is detected with alterations in a sample (*s*), *M_gs_* is set to 1; otherwise, *M_gs_* is set to 0. B. The miRNAs that are associated with ovarian cancer prognosis were identified using Cox regression analysis. All of the ovarian cancer samples were divided into three groups: the *BRCA* 1/2 altered group (*BRCA* 1/2 alteration carriers), the miRNA-related high-risk group and the miRNA-related low-risk group. Survival difference and differential expression among the groups were then assessed.

**Figure 2:**
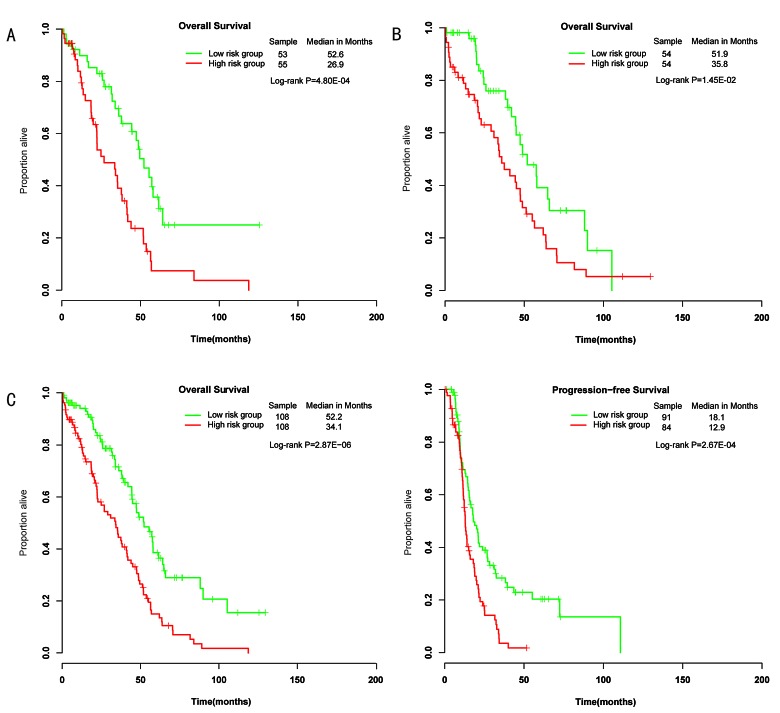
Differences in overall survival and progression-free survival were assessed between the miRNA-related low-risk and high-risk groups A, The training set. B, The testing set. C, The entire wild-type *BRCA1/2* set.

### Validation of the BRCA1/2-directed miRNA signature in the testing set and the entire wild-type *BRCA1/2* set

**Figure 3:**
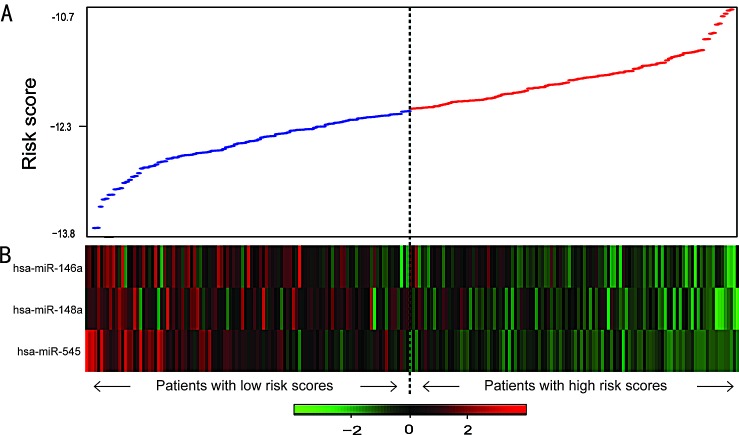
miRNA risk score analysis of ovarian cancers with wild-type *BRCA1/2* A, miRNA risk score distribution. B, Heatmap of the miRNA expression profiles. Rows represent miRNAs, and columns represent patients. The black dotted line represents the median risk score cutoff dividing patients into miRNA-related low-risk and high-risk groups.

To confirm our findings, we validated the BRCA1/2-directed miRNA signature in the testing set. Using the risk score formula, we classified patients in the testing set into a high-risk group (n=54) and a low-risk group (n=54) using the same cutoff as in the training set. Consistent with our findings in the training set, patients in the low-risk group had a significantly longer median overall survival than did those in the high-risk group (*P*=1.45E-02, median OS= 51.9 vs 35.8 months) (Figure 2B). Combining the training and testing sets, the entire cohort of patients with wild-type *BRCA1/2* also yielded similar results (*P*=2.87E-06, median OS= 52.2 vs 34.1 months; *P*=2.67E-04, median PFS=18.1 vs 12.9 months; Figure 2C). The distribution of miRNA risk score and miRNA expression is shown in Figure 3. In the entire wild-type *BRCA1/2* set, the miRNAs were expressed at significantly higher levels in the low-risk patients than in the high-risk patients (*P*<0.05).

### Independence of prognostic value of the BRCA1/2-directed miRNA signature from other clinical variables

We investigated whether the prognostic value of the BRCA1/2-directed miRNA signature was independent of other clinical variables. The univariate and multivariate Cox analysis consistently revealed that only the BRCA1/2-directed miRNA signature risk score and the treatment response were significantly associated with OS (*P*<0.05) (Table 2). Next, a data stratification analysis was performed according to treatment response, which stratified the wild-type *BRCA1/2* patients into a complete response (CR) group and a non-CR group, which included patients with a partial response, stable disease and progressive disease. The risk score of the three-miRNA signature could further subdivide patients with a CR into groups with significantly different survival times (*P*=0.01, median OS= 32.0 vs 18.5 months, log-rank test; Supplementary Figure 2). Similarly, even among those patients with a non-CR, the risk score could be used to separate patients into two subgroups with significantly different survival times (*P*=0.01, median OS= 61.5 vs 48.6 months, log-rank test) (Supplementary Figure 2). These results suggest that the BRCA1/2-directed miRNA signature is an independent prognostic factor for ovarian cancer with wild-type *BRCA1/2*.

**Table 2:** Univariate and multivariate Cox regression analysis

Variables	Univariate model		Multivariate model	
	Hazard Ratio (95% CI)	*P* Value	Hazard Ratio (95% CI)	*P* Value
3-miRNA signature risk score	1.80 (1.34 to 2.42)	1.04E-04	2.04 (1.40 to 2.98)	2.31E-04
Age (<=59.58 vs >59.58)	1.10 (0.77 to 1.56)	0.61	1.2240 (0.79 to 1.90)	0.37
Stage	1.29 (0.90 to 1.86)	0.16	1.20 (0.75 to 1.91)	0.45
Grade	1.89 (0.96 to 3.74)	0.07	2.04 (0.96 to 4.30)	0.06
Treatment response (CR vs non-CR)	0.27 (0.18 to 0.41)	8.65E-10	0.24 (0.15 to 0.38)	1.93E-09
Subtype	0.93(0.8 to 1.09)	0.38	0.13 (0.94 to 1.39)	0.18

### Prognostic differences among the *BRCA1/2* alteration carriers, and miRNA-related high-risk and low-risk patients

**Figure 4:**
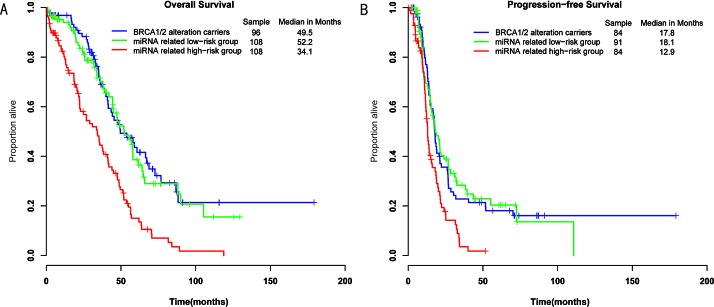
Differences in overall survival and progression-free survival were assessed among the three groups A, The log-rank *P* value of overall survival for the miRNA-related low-risk group vs the miRNA-related high-risk group (*P*=2.87E-06); the BRCA-altered group vs the miRNA-related low-risk group (*P*=0.69); the BRCA-altered group vs the miRNA-related high-risk group (*P*=8.54E-07). B, The log-rank *P* value of progression-free survival for the miRNA-related low-risk group vs the miRNA-related high-risk group (*P*=2.70E-04); the BRCA-altered group vs the miRNA-related low-risk group (*P*=0.82); the BRCA-altered group vs the miRNA-related high-risk group (*P*=6.90E-04).

The 317 ovarian cancer patients were divided into three groups: the *BRCA1/2* alteration carriers group, the miRNA-related high-risk group and the miRNA-related low-risk group. We tested pairwise comparisons of OS and PFS between the groups using the log-rank test. The miRNA-related low-risk group had a significantly better survival than those in the miRNA-related high-risk group (*P*=2.87E-06, median OS= 52.2 vs 34.1 months; *P*=2.67E-04, median PFS= 18.1 vs 12.9 months; Figure 4). Additionally, the *BRCA1/2* alteration carriers had a significantly longer survival than did patients in the miRNA-related high-risk group (*P*=8.54E-07, median OS= 49.5 vs 34.1 months; *P*=6.90E-04, median PFS= 17.8 vs 12.9 months; Figure 4). The *BRCA1/2* alteration carriers showed no significant difference in prognosis from patients in the miRNA-related low-risk group (*P*=0.69, median OS= 49.5 vs 52.2 months; *P*=0.82, median PFS= 17.8 vs 18.1 months; Figure 4). In other words, patients with wild-type *BRCA1/2* and miRNA deregulation also had a better prognosis. These results indicate that patients with either alterations in *BRCA1/2* or deregulation in miRNAs targeting *BRCA1/2* have a good prognosis and may share a common mechanism, in which miRNAs regulate the DNA repair-related pathway by targeting *BRCA1/2*.

### MiRNAs and *BRCA1/2* expression in ovarian cancers

**Figure 5:**
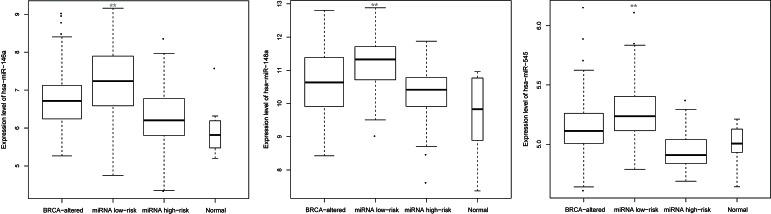
The expression of three BRCA1/2-directed miRNAs in ovarian cancer cases and normal cases BRCA-altered refers to the group of *BRCA1/2* alteration carriers. miRNA low-risk and miRNA high-risk refer to the patients with wild-type *BRCA1/2* who are predicted to fall into the high-risk and low-risk groups, respectively, according to the BRCA1/2-directed miRNA signature. ** means *P*<0.01.

According to the miRNA-target regulation data from the databases (see *Methods*), hsa-miR-146a is predicted to target *BRCA1* and *BRCA2*; hsa-miR-545 is predicted to target *BRCA1*; and hsa-miR-148a is predicted to target *BRCA2* (Supplementary Figure 3). We found that the three miRNAs were significantly up-regulated in the miRNA-related low-risk group compared with the miRNA-related high-risk group, the *BRCA1/2* alteration carriers, and the normal samples (*P*<0.01) (Figure 5). There were no significant differences in the expression values of the three miRNAs between the miRNA-related high-risk group and the normal samples (*P*=0.39 for hsa-miR-545, *P*=0.17 for hsa-miR-148a and *P*=0.25 for hsa-miR-146a). Additionally, there were no significant differences in the expression of these miRNAs between the *BRCA1/2* alteration carriers and the normal samples (*P*=0.076 for hsa-miR-545, *P*=0.065 for hsa-miR-148a and *P*=0.022 for hsa-miR-146a). Detailed results are shown in Supplementary Table 2. Those results suggested that the three miRNAs were deregulated in a portion of the wild-type *BRCA1/2* cases and that this deregulation might have facilitated the good prognosis of these ovarian cancers.

Based on the above analysis, we combined the *BRCA1/2* alteration carriers and miRNA-related low-risk patients into a good prognosis group, and the miRNA-related high-risk patients were defined as the poor prognosis group. We found that although the genes *BRCA1* and *BRCA2* trended toward being down-regulated in the miRNA-related low-risk group compared with the poor prognosis group, this fell short of significance. Nevertheless, the expression values of *BRCA1* and *BRCA2* were significantly lower in the good prognosis group than in the poor prognosis group (*P*=1.90E-05 for *BRCA1* and *P*=1.60E-02 for *BRCA2*). These results indicate that the three miRNAs may facilitate a good prognosis for ovarian cancer by down-regulating *BRCA1/2* and deregulating the DNA repair-related pathways.

### Enrichment analysis of DNA damage response pathways

Deregulation of BRCA1/2-directed miRNA and alteration of *BRCA1/2* may share a common mechanism that affects the prognosis of ovarian cancer patients treated with platinum-based chemotherapy. Based on this hypothesis, we first detected differentially expressed genes (DEGs) between the miRNA-related low-risk group and the miRNA-related high-risk group (miRNA-DEGs) and between the *BRCA1/2* alteration carrier group and the miRNA-related high-risk group (BRCA-DEGs), respectively. We found that 98% of overlapping genes between the two DEG lists (miRNA-DEGs and BRCA-DEGs) were highly consistent in their deregulation directions (up-regulation or down-regulation). Next, using functional enrichment analysis, we further investigated whether the DEGs participated in the DNA repair-related biological processes derived from the GO database. The results revealed that the genes in the two DEG lists were significantly enriched in the DNA repair-related pathways (Supplementary Table 3) (*P*<0.05). The pathways most enriched in BRCA-DEGs were “DNA damage response, signal transduction by p53 class mediator resulting in cell cycle arrest” (GO: 0006977, *P*=8.74E-05) and “signal transduction involved in mitotic G1 DNA damage checkpoint” (GO: 0072431, *P*=8.74E-05). The pathway most enriched in the miRNA-DEGs was “DNA damage response, signal transduction by p53 class mediator” (GO: 0030330, *P*=2.30E-03). Some pathways, such as “signal transduction in response to DNA damage” (GO: 0042770) and “DNA damage response, signal transduction by p53 class mediator” (GO: 0030330), were enriched in both the BRCA-DEGs and miRNA-DEGs. These results suggested that similar to *BRCA1/2* alterations, the deregulation of miRNAs affected the prognosis of ovarian cancers by regulating DNA damage response-related pathways.

## DISCUSSION

*BRCA1* and *BRCA2* function at different stages of the DNA damage response and DNA repair but act in concert to protect the genome from double-stranded DNA damage during DNA replication[7]. Our results confirmed the findings that 30% of ovarian cancer patients carrying alterations in *BRCA1/2* had a better prognosis than *BRCA1/2* alteration non-carriers. However, a proportion of patients with wild-type *BRCA1/2* may also have favorable prognosis as a result of other mechanisms, such as the deregulation of miRNAs. In this study, an analysis of 218 high-grade serous ovarian cancer cases with wild-type *BRCA1/2* revealed a BRCA1/2-directed miRNA signature model. The deregulation of the three miRNAs (hsa-miR-545, hsa-miR-146a and hsa-miR-148a) was significantly associated with favorable OS and PFS in wild-type *BRCA1/2* ovarian cancer patients. By analyzing the expression profiles and GO functional enrichment, we unraveled that carriers of *BRCA1/2* alterations and patients with miRNA deregulation shared a common mechanism that affected the prognosis of ovarian cancer treated with platinum-based chemotherapy. In other words, these miRNAs also participated in the DNA damage response and repair-related pathways by regulating *BRCA1/2*. In summary, our work identified that some *BRCA1/2* alteration non-carriers benefit from platinum-based chemotherapy, a finding that has potentially important implications for the clinical management of patients with ovarian cancer.

Kang *et al.* showed that some genes that are differentially expressed between ovarian cancer patients with poor and favorable outcomes are involved in the repair of platinum-induced DNA damage and extracted 151 DNA repair-related genes from the literature[23]. The three miRNAs (hsa-miR-545, hsa-miR-146a and hsa-miR-148a) that we identified targeted 29 of these 151 DNA repair genes, and their regulatory relationships were constructed as a network (Supplementary Figure 3). Six of the network genes were significantly down-regulated in the miRNA-related low-risk group compared with the miRNA-related high-risk group. These results indicate that the three miRNAs target not only *BRCA1* or *BRCA2* but also multiple DNA repair pathway genes to response platinum-based chemotherapy.

Some studies have reported that miRNAs can impact sensitivity to cancer therapy by targeting *BRCA1/2*. Moskwa *et al.* found that the miR-182-mediated down-regulation of *BRCA1* affected DNA repair and sensitivity to inhibitors of poly (ADP-ribose) polymerase 1 in breast cancer cell lines[21]. miR-9 down-regulated *BRCA1* and impeded DNA damage repair in ovarian cancer cells, which could improve chemotherapeutic efficacy[22]. Some previous studies have confirmed the relationships between hsa-miR-146a and *BRCA1* and between hsa-miR-545 and *BRCA1*[24, 25]. Our future work will focus on validating the miRNA-target relationships among hsa-miR-146a, hsa-miR-148a and *BRCA2* and exploring the effect of deregulation of miRNAs on sensitivity to platinum-based chemotherapy using wet experiments on cell lines and clinical samples. Despite the functional evidence that we have presented, one limitation of our study is the lack of an independent multidimensional dataset to validate our conclusion. Nevertheless, the discovery that miRNAs represent another mechanism that affects the prognosis of ovarian cancer patients with wild-type *BRCA1/2* may have important implications for clinical prediction and trial design. Moreover, long non-coding RNAs (lncRNAs), which are non-protein-coding transcripts ranging from 200 nucleotides (nt) to ~100 kilobases (kb) in length[26], are frequently aberrantly expressed in cancers and can be used for cancer diagnosis and prognosis[27, 28]. However, the roles of lncRNAs in regulating *BRCA1/2* and their impact on the prognosis of ovarian cancer have yet to be elucidated, and these warrant detailed research in the future.

## MATERIALS AND METHODS

### Datasets and processing

317 high-grade serous ovarian adenocarcinomas and 8 normal fallopian tube samples, including all information on mRNA expression, miRNA expression, mutation, promoter methylation, DNA copy number and clinical features, were downloaded from the TCGA data portal[2]. The level 3 mutation profile (somatic mutation and germline mutation information) was used. The level 1 copy number data were processed using the dChip software, the GLAD algorithm and the GISTIC algorithm, as described by Gu *et al*[29]. Level 3 IlluminaInfinium DNA methylation data were processed as described by Yang *et al*[30]. By integrating the discrete mutation profile, copy number alteration profile and methylation profile, we obtained the gene alteration profile (*M_gs_*), in which the columns reflect ovarian cancer samples and the rows reflect genes. If a gene (*g*) was detected with alterations in a sample (*s*), we set *M_gs_* to 1; otherwise, *M_gs_* was set to 0 (Figure 1A). Here, alterations in the *M_gs_* included somatic mutations, germline mutations, copy number amplifications/deletions and hypo/hyper-methylations. All patients received platinum-based chemotherapy after surgery. The detailed clinical features of the patients are listed in Table 1.

Considering that miRNA targets predicted by multiple algorithms might be more reliable, we used miRNA-target interactions appearing in at least two of nine databases: TargetScan[31], miRanda[32], PicTar[33], miRBase[34], DIANA-microT[35], PITA[36], miRNAMap[37], miRTarBase[38] and miRecords[39].

### Identification of predictive BRCA1/2-directed miRNA signature

In total, germline and somatic mutations, hypermethylations or deletions in the genes *BRCA1* or *BRCA2* (Supplementary Table 1) were detected in 99 patients. The 218 samples with wild-type *BRCA1/2* were randomly divided into a training set (n=109 samples) and a testing set (n=109 samples). First, in the training set, the association between the miRNAs targeting *BRCA1/2* and survival was assessed by univariate Cox regression analysis (Figure 1B). Second, a miRNA prediction classifier was constructed by linear combination of the expression values of the BRCA1/2-directed miRNAs and the Cox regression coefficient as the weight. The patients were classified into high-risk and low-risk groups by utilizing the median risk score as the cutoff point (low-risk group was 1, high-risk group was 0). Then, the log-rank test was used to assess the overall survival between the low-risk and high-risk groups. In the Cox analysis, miRNAs with *P*<0.05 were selected as signature that were significantly associated with the OS of ovarian cancer. Finally, we validated the miRNA risk-prediction model in the testing set. The 317 ovarian cancer samples from the TCGA data portal were divided into three groups: the *BRCA1/2* alteration carrier group, the miRNA related high-risk group and the miRNA related low-risk group. Kaplan-Meier survival plots and the log-rank test were used to assess the differences in OS and PFS among the three groups. Multivariate Cox analysis was applied to test whether the BRCA1/2-directed miRNA signature was independent of other clinical characteristics such as age, tumor grade and so on.

### Statistical analysis

A two-sample t-test was performed to identify the differentially expressed miRNAs or mRNAs in the level 3 miRNA expression profile and level 3 mRNA expression profile, respectively. The hypergeometric distribution model was used to test whether the DNA repair-related terms from the Gene Ontology (GO) database[40] were significantly enriched in the differentially expressed genes[41].

## SUPPLEMENTARY MATERIAL, FIGURES AND TABLES



## Abbreviations

CR: Complete Response
DEG: Differential Expression Gene
HR: Hazard Ratio
OS: Overall Survival
PFS: Progression-Free Survival
GO: Gene Ontology
