# Supplementary material for: The BRCA1/2-directed miRNA signature predicts a good prognosis in ovarian cancer patients with wild-type BRCA1/2
Source: Oncotarget. 2014 Dec 11;6(4):2397–406. doi: 10.18632/oncotarget.2963 (PMC4385859; doi:10.18632/oncotarget.2963)
Supplement: Supplementary file 1 [file oncotarget-06-2397-s001.pdf]

The BRCA1/2-directed miRNA signature predicts a good prognosis in ovarian cancer patients with wild-type BRCA1/2

Supplementary Material

Supplementary Table 1. The number of samples with alterations in BRCA1 and BRCA2

Symbol	Germline mutation	Somatic mutation	Hypermethylation	Copy number deletion
BRCA1	27	10	31	1
BRCA2	20	13	0	0

Supplementary Table 2. Comparison of the expression values of three miRNAs between different ovarian cancer groups.

	Hsa-miR-146a		Hsa-miR-148a		Hsa-miR-545	
	P	Direction	*P*	Direction	*P*	Direction
miRNA low-risk vs miRNA high-risk	8.45E-13	up	5.45E-16	up	2.20E-16	up
miRNA low-risk vs BRCA-altered	2.07E-04	up	1.47E-06	up	5.94E-05	up
BRCA-altered vs Normal	0.02	/	0.07	up	0.08	/
miRNA low-risk vs Normal	1.90E-03	up	9.40E-03	up	2.70E-03	up
miRNA high-risk vs Normal	0.25	/	0.17	up	0.39	/

Note: BRCA-altered refers to the group of *BRCA1/2* alteration carriers. miRNA low-risk and miRNA high-risk refer to the patients with wild-type *BRCA1/2* who are predicted to fall into high-risk and low-risk groups by the miRNA signature. Direction refers to the up-regulation (up) or down-regulation (down) of significantly differentially expressed genes between the two groups.

Supplementary Table 3. GO enrichment analysis of the differentially expressed genes.

GO ID	Term name	*P*
**BRCA-altered vs miRNA high-risk**		
GO:0006977	*DNA damage response, signal transduction by p53 class mediator resulting in cell cycle arrest*	8.74E-05
GO:0072431	*signal transduction involved in mitotic G1 DNA damage checkpoint*	8.74E-05
GO:0072422	*signal transduction involved in DNA damage checkpoint*	1.08E-04
GO:0042770	*signal transduction in response to DNA damage*	2.28E-04
GO:0031571	*mitotic G1 DNA damage checkpoint*	4.18E-04
GO:0044783	*G1 DNA damage checkpoint*	4.18E-04
GO:0044773	*mitotic DNA damage checkpoint*	1.32E-03

GO:0030330	*DNA damage response, signal transduction by p53 class mediator*	3.12E-03
GO:0000077	DNA damage checkpoint	9.66E-03
GO:0070914	UV-damage excision repair	1.99E-02
GO:0045739	positive regulation of DNA repair	2.40E-02
GO:2001020	regulation of response to DNA damage stimulus	3.97E-02
**miRNA low-risk vs miRNA high-risk**		
GO:0030330	*DNA damage response, signal transduction by p53 class mediator*	2.31E-03
GO:0008630	intrinsic apoptotic signaling pathway in response to DNA damage	4.93E-03
GO:0042770	*signal transduction in response to DNA damage*	5.18E-03
GO:0043518	negative regulation of DNA damage response, signal transduction by p53 class mediator	8.39E-03
GO:1902165	regulation of intrinsic apoptotic signaling pathway in response to DNA damage by p53 class mediator	8.39E-03
GO:1902166	negative regulation of intrinsic apoptotic signaling pathway in response to DNA damage by p53 class mediator	8.39E-03
GO:1902230	negative regulation of intrinsic apoptotic signaling pathway in response to DNA damage	1.02E-02
GO:1902229	regulation of intrinsic apoptotic signaling pathway in response to DNA damage	1.21E-02
GO:0006977	*DNA damage response, signal transduction by p53 class mediator resulting in cell cycle arrest*	1.36E-02
GO:0072431	*signal transduction involved in mitotic G1 DNA damage checkpoint*	1.36E-02
GO:0072422	*signal transduction involved in DNA damage checkpoint*	1.47E-02
GO:0031571	*mitotic G1 DNA damage checkpoint*	2.49E-02
GO:0044783	*G1 DNA damage checkpoint*	2.49E-02
GO:0000731	DNA synthesis involved in DNA repair	3.23E-02
GO:0045008	depyrimidination	3.91E-02
GO:0044773	*mitotic DNA damage checkpoint*	3.93E-02
GO:2001021	negative regulation of response to DNA damage stimulus	4.60E-02
GO:0000718	nucleotide-excision repair, DNA damage removal	4.64E-02

Note: BRCA-altered refers to the group of *BRCA1/2* alteration carriers. miRNA low-risk and miRNA high-risk refer to the patients with wild-type *BRCA1/2* who are predicted to fall into high-risk and low-risk groups by the miRNA signature.The overlapping GO terms in which both lists of differentially expressed genes were enriched are marked in italics. $P<0.05$ was considered to be statistically significant.

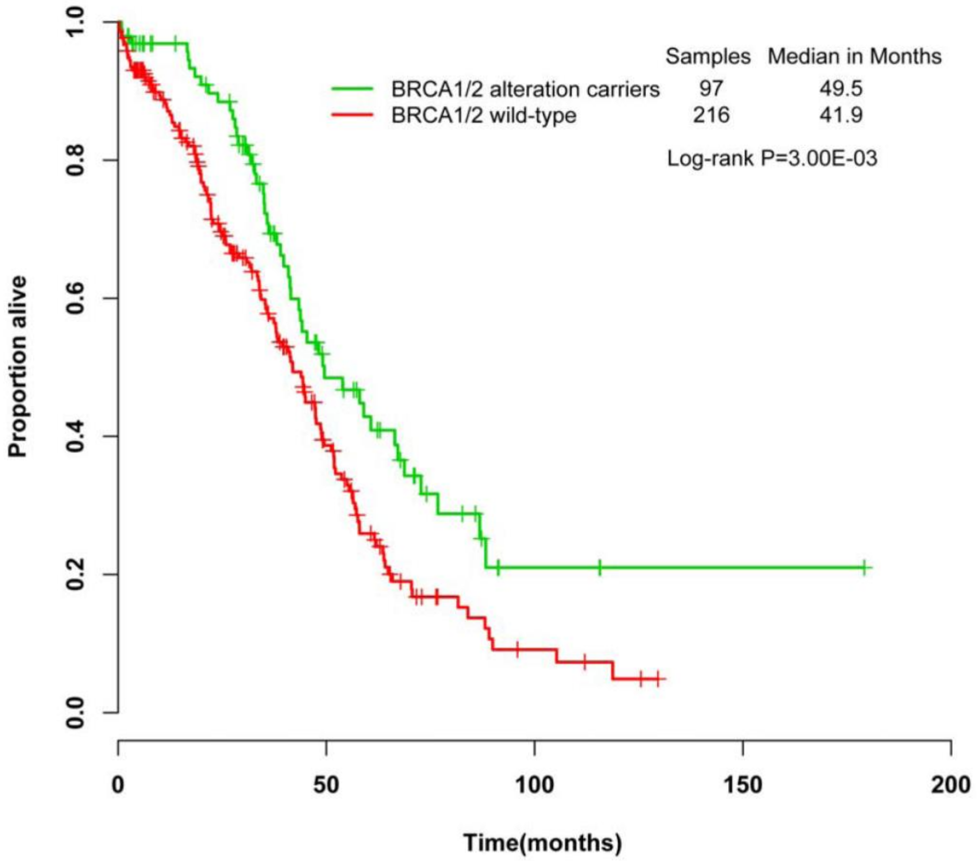

Supplementary Figure 1. Difference in overall survival was assessed between *BRCA1/2* alteration carriers and non-carriers.

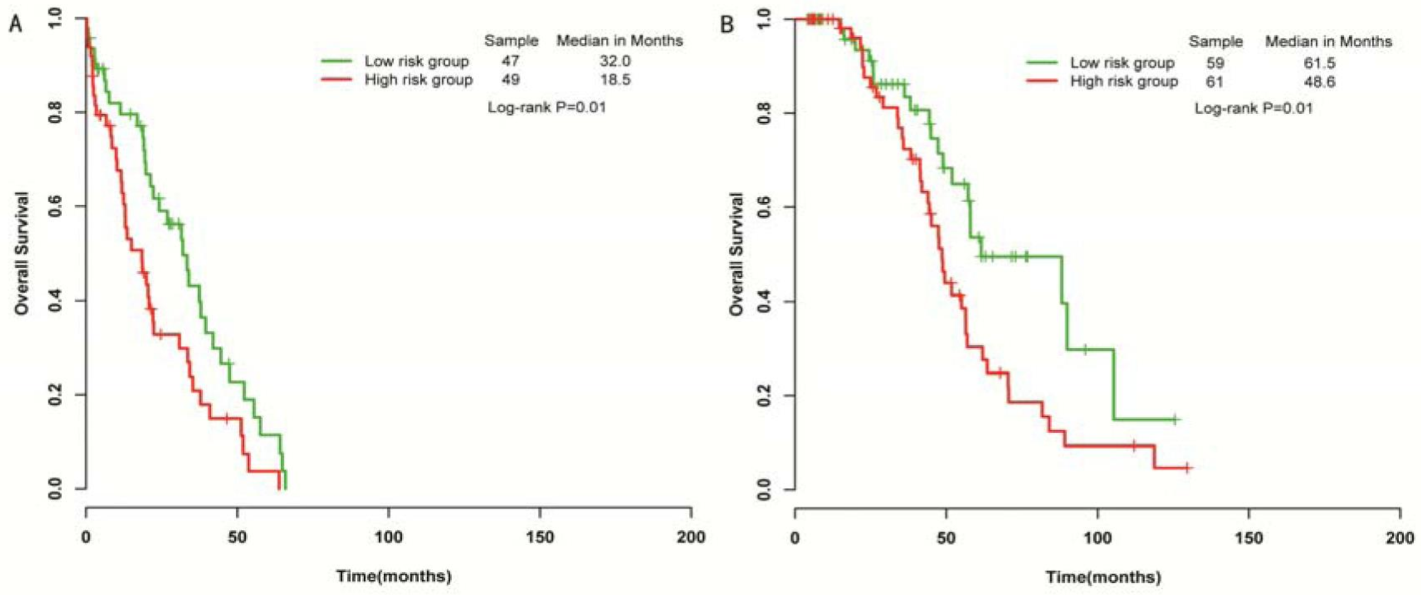

Supplementary Figure 2. Difference in overall survival was assessed between the low-risk group and high-risk groups. A, patients with CR status. B, patients with non-CR status.

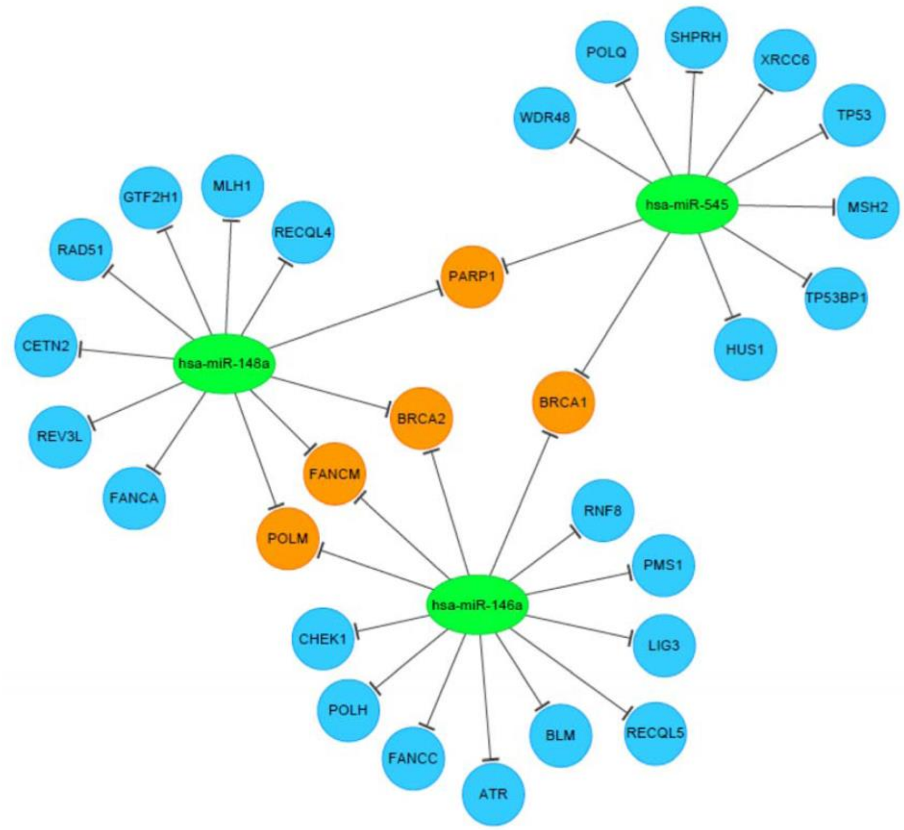

Supplementary Figure 3. miRNA and DNA repair gene regulation network. Ellipses in green represent miRNAs. Blue circles represent target genes. Blocking arrows depict the relationship between miRNAs and their target genes. Yellow circles represent genes that are targeted by more than one miRNA.
